# Meaning-centered therapy in Ukraine's war refugees: An attempt to cope with the absurd?

**DOI:** 10.3389/fpsyg.2022.1067191

**Published:** 2022-12-22

**Authors:** Alessandra Costanza, Andrea Amerio, Andrea Aguglia, Luca Magnani, Philippe Huguelet, Gianluca Serafini, Maurizio Pompili, Mario Amore

**Affiliations:** ^1^Department of Psychiatry, Faculty of Medicine, Geneva University (UNIGE), Geneva, Switzerland; ^2^Department of Psychiatry, Adult Psychiatry Service (SPA), University Hospitals of Geneva (HUG), Geneva, Switzerland; ^3^Department of Neuroscience, Rehabilitation, Ophthalmology, Genetics, Maternal and Child Health (DINOGMI), Section of Psychiatry, University of Genoa, Genoa, Italy; ^4^IRCCS Ospedale Policlinico San Martino, Genoa, Italy; ^5^Department of Neurosciences, Mental Health and Sensory Organs, Suicide Prevention Center, Sant'Andrea Hospital, Sapienza University of Rome, Rome, Italy

**Keywords:** war, Ukraine, refugees and asylum seekers, children, mental health and psychosocial programs, demoralization, meaning in life, meaning-based psychotherapy

## The current situation in Ukraine and mental health consequences

The Russian invasion of Ukraine started on February 24, 2022, and is still ongoing, causing the largest civilian refugee crisis in Europe since World War II, and the first of its kind since the Yugoslav war in the 1990's. At the time of writing, the United Nations High Commissioner for Refugees (UNHCR) estimated that more than 14 million people had either left the country or been displaced internally [United Nations High Commissioner for Refugees (UNHCR), 2022a,b]. The most vulnerable population segments among refugees are children (more than half of all Ukrainian children have been forced to leave their homes), women, elder persons, and those who are ill and unable to participate in the military response (Dobson, 2022; Hodes, 2022). These individuals are at a very high risk of developing a wide range of mental health disorders, including severe anxiety and depressive symptoms, post-traumatic stress disorder (PTSD), and suicidal ideation/behavior with likely long-term sequelae (Charlson et al., 2019; Javanbakht, 2022; Elvevåg and DeLisi, 2022). Johnson et al. (2022), conducting face-to-face interviews while the war was still confined to the Donbass and Kharkiv regions, found widespread direct exposure to conflict-related traumatic events (65%) among internally displaced people (IDP) leading to an elevated prevalence of PTSD symptoms across all socio-demographic groups. Similar results were found by Roberts et al. (2019) who used a cross-sectional survey on IDP during the early stages of the conflict and found prevalences of PTSD, depression, and anxiety of 32, 22, and 17%, respectively. Cheung et al. (2019) noted that more than half the IDP in Ukraine were at risk of somatic distress (55%). All this must be seen in the context of a public health care system that is under increased pressure due to the ongoing Russian targeting of residential areas and crucial civilian infrastructures. This had already created a considerable treatment gap in 2017 when 74% of individuals who had either screened positive with PTSD, depression, or anxiety or self-reported a problem were unable to receive Mental Health and Psychosocial Support (MHPSS) (Roberts et al., 2019).

## Resources available to refugees and health care professionals

The American Psychiatric Association (APA) pointed out that specific attention should be paid to the presence and intensity of signs indicative of psychiatric/psychological suffering in refugee populations, irrespective of whether they pertain to an already established pathology or prodrome [American Psychiatric Association (APA), 2022]. Therefore, comprehensive MHPSS programs for refugees that integrate somatic health concerns, social support, education, and targeted psychiatric/psychological interventions are urgently needed (Murphy et al., 2022). A broad range of resources can be used to address and alleviate the war's impact on mental health from a primarily pragmatic and easy-access point of view, including various types of psychological and psychotherapeutic strategies that can be applied to these populations (Shevlin et al., 2022; Uphoff et al., 2020). In Table 1 we have assembled (according to our team research consensus) a summary of some of the resources available to refugees and healthcare providers, which we found useful and consider pertinent, including guidelines for various emergency care situations and refugees.

**Table 1:** Examples of resources available to refugees and healthcare professionals to provide mental health support (non-exhaustive).

**Target audience**	
**Refugees**	**Healthcare providers**
Help in Ukraine and neighboring countries: UNHCR: https://help.unhcr.org/switzerland/ukraine/help-in-ukraine-and-neighboring-countries/ https://help.unhcr.org/	“*APA statement and resources on the mental health impact of the war in Ukraine,” 2022:* https://www.psychiatry.org/news-room/news-releases/apa-statement-and-resources-on-the-mental-health-i
Online and face-to-face psychological support for teenagers and parents: PORUCH: https://poruch.me/	“*Mental health and psychosocial support in the way of a humanitarian response in Ukraine and neighboring countries,” 2022:* https://www.pscentre.org/wp-content/uploads/2022/03/resources-ukraine17.pdf
Psychological consultations in Ukrainian via phone: TELL ME: https://tellme.com.ua/	“*Inter-Agency Standing Committee (IASC) guidelines on mental health and psychosocial support in emergency settings,” 2007:* https://www.who.int/publications/i/item/iasc-guidelines-for-mental-health-and-psychosocial-support-in-emergency-settings
Online platform to find psychological help and practical information during war time: VIYNA: https://viyna.net/	“*Mental health and psychosocial support for refugees, asylum seekers, and migrants on the move in Europe. A multi-agency guidance note,” 2015:* https://www.who.int/publications/i/item/mental-health-and-psychosocial-support-for-refugees-asylum-seekers-andmigrants-on-the-move-in-europe
	Links to mental health and psychological support and mental health assessment materials in several languages: The International Trauma Consortium: https://www.traumameasuresglobal.com/ukraine

## War refugees dedicated interventions and their effectiveness

Before any intervention can take place, an initial screening needs to be conducted where the immediate focus should be placed on questions to identify serious mental illness and risk for suicide before proceeding with a more formal mental health assessment once rapport has been established [Hollifield et al., 2012; Centers for Disease Control and Prevention (CDC), 2022]. Women should also be screened for possible sexual abuse to guide interventions (Ekblad et al., 2007).

About conflict settings, in general, there is little guidance on how to deliver mental health interventions that are suitable (Slobodin and de Jong, 2015; Gaffey et al., 2021), especially during the active phase of war (Martsenkovskyi et al., 2022). Acarturk et al. (2022), assessing the effectiveness of a WHO self-help psychological intervention for preventing mental disorders among Syrian refugees in Turkey, found that although the self-help approach was not effective immediately post-intervention participants enrolled in the self-help program were significantly less likely to have any mental disorders and also saw beneficial effects in terms of depression and quality of life at the six-month follow-up compared to those in the enhanced care as usual group. Based on a meta-analysis of interventions designed specifically for traumatized asylum seekers and refugees, Slobodin and de Jong (2015) concluded that cognitive-behavioral therapy (CBT) and narrative exposure therapy (NET) were two evidence-based strategies that proved effective and suitable for refugee populations. They did not find sufficient data to confirm or refute alternative approaches, although there appears to be some preliminary evidence that a combination of eye movement desensitization and reprocessing (EMDR) and stabilization provided positive outcomes. In a similar meta-analysis, Turrini et al. (2019) also found that CBT was effective at reducing PTSD and anxiety while EMDR was effective with depressive symptoms. In contrast, narrative exposure therapy (NET) proved ineffective. They authors further conclude that most studies were conducted in adults or mixed populations of adults and children, which makes the efficacy of these psychosocial interventions in children uncertain.

Concerning refugees, and refugee vulnerable people in particular, data on effective psychotherapeutic interventions are extremely limited (Peltonen and Punamäki, 2010; Pacione et al., 2013). There are some smaller studies that have shown that both Narrative Exposure Therapy (NET) and a version specifically adapted to your (KidNET) have been used successfully to treat refugee youth suffering from symptoms of PTSD (Schaal et al., 2009; Robjant and Fazel, 2010; Ruf et al., 2010) and more detailed studies are underway (Schwartz et al., 2022; Velu et al., 2022).

Clearly, defining a single set of universally valid interventions appropriate in all cultural contexts is an impossibility as interventions are typically only culturally appropriate for those settings in which they have been developed (Elvevåg and DeLisi, 2022; Gaffey et al., 2021). Globally, it seems that there is no a real consensus regarding the efficiency of these interventions, particularly for psychological/psychotherapy strategies.

In this opinion letter, we intend to describe our initial experience in caring for refugees from the war in Ukraine using a meaning-based psychotherapy approach.

## Caring for Ukrainian refugees: From demoralization to meaning-centered psychotherapy

At our mental health service we focused on a conceptual model that combines the two psychological constructs of demoralization and meaning in life (MiL), which are closely linked (loss of MiL being a sub-component of demoralization) (Clarke and Kissane, 2002).

Of note, these constructs were conceived specifically for populations who suffered a dramatic fracture that divided their existence into a “before” and “after” in wartime contexts concerning civilian refugees, concentration camp survivors, soldiers, and veterans. Demoralization was first described in American soldiers confronted by an unfamiliar disease (Frank, 1974), and the attribution/preservation of MiL, even if relative and transient, was first studied in concentration camp survivors as a possible key factor for survival while being exposed to unavoidable and incomprehensible atrocities (Frankl, 1959). After these initial conceptualizations, various models of demoralization and MiL have been proposed for the purpose of being used in clinical practice as risk and resilience factors, respectively, in heterogeneous populations, without or with a psychiatric diagnosis, e.g., in perpective of recovery's encouragement or to refine the assessment of suicidal risk (which represents a common final path for many forms of suffering: psychic, somatic, and psychosomatic; it is particularly in this latter domain where these two constructs have been researched and applied most widely) (Huguelet et al., 2016; Costanza et al., 2020a,b).

As part of our institutional mental health support activities, we organized periodic therapeutic sessions with Ukrainian women, who, along with their children, had arrived in Italy as refugees. The therapeutic sessions were conducted in groups with ten participants on average and accompanied by the same interpreter. A full cycle includes four weekly sessions (we chose this 1-month duration as refugees arriving in Italy typically only stay for one month in their first location before being transferred to another, more long term site). Each session within a cycle was co-led by the same psychiatrist and psychologist.

Although in this population a clear diagnosis of depression according to the Diagnostic and Statistical Manual of Mental Disorders - Fifth Edition (DSM-V) is often difficult to establish, what do emerge prominently are the themes representing the five sub-components of demoralization, namely loss of MiL, hopelessness or disheartenment, helplessness, sense of failure, and dysphoria. Hence, since loss of MiL is a crucial sub-component of demoralization, we conducted these group interventions inspired by meaning-centered therapy.

The theory and rationale underlying the psychotherapeutic model used in our sessions are based on meaning-centered coping strategies. In the late 1990's, Folkman added meaning-centered coping techniques to the original emotion- or problem-based coping strategies of Lazarus by extending the resolution pathway to include the impacts of resources experienced by individuals as they encountered unfavorable outcomes (Folkman, 1997). Meaning-focused coping was defined as “appraisal-based coping in which the person draws on his or her beliefs (e.g., religious, spiritual, or justice-related), values, and existential goals (e.g., purpose in life, guiding principles) to motivate and sustain coping during a difficult time” (Janoff-Bulman, 1992). Severe trauma can represent a kind of ontological assault in which some of the most fundamental assumptions held by the individuals (mentioned above), including that the world is benevolent and meaningful and the self is worthy (Janoff-Bulman, 1992), are shattered, potentially leading to the dissolution of one's personal biography, self-identity, and perceived world. The post-traumatic crisis can imply a challenge to maintaining MiL, with the acquisition of a peculiar depressive disposition (*Befindlichkeit*) well-captured by the dimensions of demoralization (Janoff-Bulman, 1992).

Interventions were transcribed verbatim in medical dossiers to be able to extract the primary themes for subsequent qualitative analysis. These included horrific accounts and images but also particular wording to describe how individuals had attempted to survive those events. Thus far, the MiL-related coping theme that emerged most strongly and most frequently was the “absolute need” to safeguard the physical and mental health of children (even in women who did not have children but who cared for those of others or orphans), according with previous observations (Costanza et al., 2020a,b). Our primary objective – in line with the link between MiL and demoralization – was a diminution of demoralization by trying to enable refugees to find subjective and effective meaning in situations where the irrational seems to prevail. Indeed, toward the end of a therapeutic session cycle, most participants would express a sense of relief and decreased levels of demoralization. This is in good agreement with a recent review on suicidal patients (community-dwellers or affected by psychiatric illnesses and severe oncological and neurological diseases) in which meaning-centered coping techniques have been postulated as useful strategies to alleviate demoralization (Costanza et al., 2022).

Agreeing with the primary instance of these mothers, we would expect that the beneficial effects this therapeutic approach had on adult refugees could help to mitigate the impact the war has had on their children (Bürgin et al., 2022; Editorial, 2022) who have been exposed to something unexpected, unpredictable, and utterly cruel.

## Conclusion and perspectives

In this opinion letter, we have provided a short overview of the available interventions for treating war refugees and their effectiveness. From a psychological/psychotherapeutic point of view, we have chosen a specific model from the extensive arsenal of possible approaches that meet APA and MHPSS recommendations for supporting refugees. We then briefly described our experience with this model, which is based on a conceptua framework that combines the two psychological constructs of demoralization and MiL.

Mental health and psychosocial support interventions are crucial as refugees are at a very high risk of developing a wide range of mental health disorders, including PTSD, severe anxiety/depressive disorders, and suicidal ideation/behavior with likely long-term sequelae. Given the magnitude of the current crisis, this requires international support from policy makers and from the institutions in the various hosting countries. We need to encourage both interpersonal support by facilitating links with compatriots in similar situations to share their concerns and experiences in their own language and host countries need to facilitate their social integration by providing language courses, opening schools to refugee children, and allowing them to take on temporary employment, etc (Kaufman et al., 2022; Schwartz et al., 2022).

Finally, while this opinion letter may be brief and somewhat anecdotal and our psychotherapeutic approach is relatively novel and perhaps even unorthodox, it has show promising results although these should be considered preliminary. We expect it to become more common once we are able to provide a more thorough analysis of the outcomes. We believe that our experience is based on solid foundations and can offer a generalizable therapeutic perspective. With the purpose of searching for and redirecting toward a personal and effective meaning where a meaning is not there and the absurd stands out. This work is necessarily in progress and must be continued.

## Author contributions

AC: conception, data collection, and composition of the initial draft. AAm: conception and major contributions to the intermediate revision of the manuscript. AAg and LM: contributions to the intermediate revision of the manuscript. PH, GS, MP, and MA: supervision and final draft revision. All authors read and approved the final manuscript.
